# Quercetagetin From *Tagetes erecta* Differentially Induces Autophagy and Ferroptosis in MDA-MB-231 and JC Breast Cancer Cells

**DOI:** 10.1155/jt/6648109

**Published:** 2025-11-12

**Authors:** L. Sánchez-Sánchez, H. López-Muñoz, O. M. Echeverría, N. Torres-Ramírez, J. J. Alvarado-Sansininea, D. Bahena-Salmerón, J. I. Martínez-Flores, N. González, M. L. Escobar

**Affiliations:** ^1^Unidad de Investigación en Diferenciación Celular y Cáncer, Laboratorio de Biología Molecular Del Cáncer, UMIEZ, Facultad de Estudios Superiores Zaragoza, Universidad Nacional Autónoma de México, Ciudad de México, Mexico; ^2^Departamento de Biología Celular, Laboratorio de Microscopía Electrónica, Facultad de Ciencias, Universidad Nacional Autónoma de México, Ciudad de México, Mexico; ^3^Facultad de Estudios Superiores Zaragoza, Universidad Nacional Autónoma de México, Ciudad de México, Mexico

**Keywords:** apoptosis, autophagy, breast cancer, ferroptosis, quercetagetin

## Abstract

Quercetagetin is a flavonoid that has shown antiproliferative effects against different human cancers. We thoroughly analysed the effects of quercetagetin obtained from *Tagetes erecta* on human triple-negative breast cancer cells (MDA-MB-231) and murine breast cancer cells (JC) to elucidate its underlying antineoplastic mechanisms. Quercetagetin exerted dose-dependent antiproliferative effects on both cell lines (half-maximal inhibitory concentration: 188 and 282 μM, respectively). While it eliminated MDA-MB-231 cells via both apoptosis and autophagy, it predominantly eliminated JC cells via ferroptosis. Our results demonstrate that quercetagetin induces different programmed cell death pathways in a species-specific manner. Notably, quercetagetin did not significantly affect the proliferation of noncancerous lymphocytic cells. These data may facilitate the development of anticancer drugs that induce programmed cell death with fewer side effects.

## 1. Introduction

The global population is impacted by different cancers. Despite diverse strategies and advances in cancer treatment, cancer incidences continue to increase worldwide. Breast cancer has reached an important milestone in the female population [1]. Conventional cancer treatments include surgery, chemotherapy and radiotherapy [2]. Chemotherapy involves treating malignant cells and tissues with chemicals; however, an important side effect of this approach is the induction of cell and tissue necrosis (reviewed in [3]).

Traditional medicine is used to treat different diseases worldwide, including cancer. Therefore, important efforts have been made to characterise the antitumour effects of diverse natural compounds [4, 5]. In particular, flavonoids are known for their biological properties, including antitumour activity [6, 7]. Indeed, in a prior study, we reported the antiproliferative effects of three flavonoids (quercetin, patuletin and quercetagetin) isolated from members of the *Tagetes* family on cancer cells [8]. Interestingly, while quercetagetin induced apoptosis, the number of dead cells did not correspond with the decrease in the total number of cells. As a result, we proposed the possible involvement of an alternative cell elimination pathway upon treatment with quercetagetin.

Several programmed and nonprogrammed cell death pathways have been described [9]. Necrosis is a nonprogrammed cell death pathway, while apoptosis, autophagy and ferroptosis are programmed cell death pathways (reviewed in [10]). Each pathway is characterised by distinct morphological and biochemical features. For example, apoptosis is characterised by cellular and nuclear compaction, DNA fragmentation and apoptotic body formation [11]. It is regulated by caspase proteases [12], which are responsible for conferring the morphological characteristics of this pathway. In contrast, autophagy is characterised by elevated cytoplasmic vesiculation, and each step of the autophagic process is carefully regulated by the autophagy-related (ATG) proteins [13].

Recently, ferroptosis has been increasingly recognised as an important cell death pathway. It is characterised biochemically by increased intracellular iron levels, reactive oxygen species generation and lipid peroxidation. Morphologically, mitochondrial damage is evident at the ultrastructural level [14]. In this study, we investigated the anticancer properties of the flavonoid quercetagetin. First, we examined its capacity to induce two programmed cell death pathways in breast cancer cell lines, observing that it induces different responses in human (MDA-MB-231) and murine (JC) breast cancer cells. We also analysed the effects of quercetagetin on noncancerous lymphocytic cells to evaluate its selectivity. Our results show that while quercetagetin eliminates human breast cancer cells via apoptosis and autophagy, it eliminates mouse breast cancer cells via ferroptosis. In contrast, quercetagetin did not impact the proliferative activity of noncancerous lymphocytic cells. Therefore, our results highlight the importance of the flavonoid quercetagetin as an inducer of programmed cell death selectively in cancer cells.

## 2. Materials and Methods

### 2.1. Compounds

Quercetagetin was isolated from *Tagetes erecta* flowers and characterised as previously described [8]. Colchicine (1 nM) (Merck, Darmstadt, Germany), rapamycin (5 nM) (Sigma-Aldrich, Saint Louis, MO) and erastin (100 nM) (Cambridge, UK) were used as positive treatments for apoptosis, autophagy and ferroptosis, respectively. Nontreated cells were included to compare the response to the treatments.

### 2.2. Isolation of Quercetagetin

500 g of *Tagetes erecta* (flower heads) was macerated at room temperature in 96% ethanol (EtOH) (1 L) for 24 h. After 24 h, the solution was filtered, and the EtOH was concentrated by reduced pressure in a rotary evaporator (BUCHI, Switzerland) until a residue of 10 g was obtained. Quercetagetin was then separated using a reverse-phase preparative plate (20 × 10 cm; 0.25 mm) (Merck, USA) eluted with a 7.8:1.2:1 ethyl acetate/methanol/water solution. Under these separation conditions, 2 g of quercetagetin was obtained. It was washed and recrystallised with ethyl acetate and methylene chloride. Purity was checked by TLC RF and melting point [15].

### 2.3. Evaluation of Cell Proliferation

Human (MDA-MB-231) and mouse (JC) breast cancer cells were purchased from the American Type Culture Collection (Rockville, MD, USA). They were cultured in the RPMI-1640 medium (GIBCO, Invitrogen Corp., Grand Island, NY, USA) supplemented with 5% foetal bovine serum (GIBCO), phenol red and benzylpenicillin in a humidified atmosphere with 5% CO_2_ at 37°C. Next, 7500 cells were seeded in 96-well tissue culture-treated plates, cultured for 24 h and treated with different doses of quercetagetin (0–100 μg/mL) for an additional 24 h. Then, the cells were fixed with 1.1% glutaraldehyde for 20 min, washed with water and stained with crystal violet for 20 min. Next, the plates were washed with water and incubated with 10% acetic acid for 20 min. Finally, the plates were read at 590 nm with an Epoch microplate spectrophotometer (Biotek, Winooski, VT, USA). The resulting data were linearised to calculate the half-maximal inhibitory concentration (IC_50_).

### 2.4. Necrosis Analysis

MDA-MB-231 and JC cells were treated with quercetagetin at their respective IC_50_s (60 μg/mL [188 μM] and 90 μg/mL [282 μM], respectively) for 24 h. Next, the cell culture supernatant was collected, and lactate dehydrogenase (LDH) activity was evaluated using an LDH Cytotoxicity Assay Kit (BioVision, Milpitas, CA, USA) according to the manufacturer's instructions. Briefly, LDH oxidises lactate to pyruvate, which then reacts with the tetrazolium salt 2-(4-iodophenyl)-3-(4-nitrophenyl)-5-phenyltetrazolium to produce formazan. The amount of formazan in the culture supernatant directly correlates with the number of lysed cells. The formazan dye is water-soluble and can be detected with a spectrophotometer at 500 nm [16].

### 2.5. Ultrastructural Evaluation

First, 1 × 10^6^ cells were seeded in 100-mm culture dishes and cultured for 24 h. Next, the medium was removed, and the cells were stimulated with quercetagetin at their respective IC_50_s for 24 h. Then, the cells were fixed with a mixture of 2.5% glutaraldehyde and 4% paraformaldehyde in phosphate-buffered saline (PBS) for 90 min and postfixed in 1% osmium tetroxide for 60 min. Next, the cells were mounted in epoxy resin (Epon814; Electron Microscopy Sciences, Hatfield, PA, USA). Then, the samples were sliced into ultrathin sections, placed on copper grids, treated with a standard contrast with 4% uranyl acetate and 0.35% lead citrate and visualised with a JEOL 1010 electron microscope (Tokyo, Japan) at 80 kV. Digital images were captured with a digital camera (Hamamatsu Photonics K.K., Shizuoka, Japan).

### 2.6. MitoTracker and Monodansylcadaverine (MDC) Assays

First, 5 × 10^5^ cells were seeded in 60-mm culture dishes containing coverslips and cultured for 24 h. Next, the cells were treated with quercetagetin at their respective IC_50_s for 24 h. Then, the coverslips were incubated in a serum-free RPMI-1640 medium containing 100 μM MDC (Sigma-Aldrich, St. Louis, MO, USA) and 200 nM MitoTracker Green FM 490⁄516 (Invitrogen, Paisley, UK) for 20 min. Finally, the cells were observed under a Nikon Eclipse E600 light microscope (Tokyo, Japan), and images were captured with a Nikon DXM1200F digital camera.

### 2.7. Immunocytochemistry

First, 5 × 10^5^ cells were seeded in 60-mm culture dishes containing coverslips and cultured for 24 h. Next, the cells were treated with quercetagetin at their respective IC_50_s for 24 h. Then, the cells were fixed in 2% paraformaldehyde in PBS (pH 7.2) and incubated with the primary antibodies listed in Table 1 overnight at 4°C. Next, the coverslips were washed with PBS and incubated with the secondary antibodies listed in Table 1 for 2 h in the dark. Finally, the cells were washed with PBS, mounted in DAPI-containing antifade medium for nuclear counterstaining (Vectashield; Vector Laboratories, Newark, CA, USA) and observed with a Nikon Eclipse E600 light microscope. Digital images were captured with a Nikon DXM1200F digital camera.

### 2.8. Identification of Neutral Lipids and Lipid Peroxidation

The cells were seeded onto coverslips for 24 h and then treated with quercetagetin at their respective IC_50_s for 24 h. Next, one coverslip was incubated in 2 μM BODIPY FL C_12_ (Invitrogen) in the RPMI-1640 medium for 20 min to identify neutral lipids, and a second coverslip was incubated with 1 μM BODIPY 581/591 C_11_ (Molecular Probes, Eugene, OR, USA) in the RPMI-1640 medium for 45 min to identify peroxidised lipids. Finally, the coverslips were washed with the RPMI-1640 medium, mounted on slides and observed under a fluorescence microscope (Nikon Eclipse E600). Digital images were captured using a Nikon DXM1200F digital camera.

### 2.9. Western Blotting

First, 1 × 10^6^ cells were seeded in 100-mm culture dishes and cultured for 24 h. Next, the medium was removed, and the cells were stimulated with quercetagetin at their respective IC_50_s for 24 h. Then, total protein was extracted by incubating the cells in RIPA lysis buffer (50 mM Tris hydrochloride [pH 7.5], 150 mM NaCl, 0.1% SDS, 1 mM PMSF, 0.5% sodium deoxycholate and 1% Nonidet P-40) supplemented with a complete protease inhibitor cocktail (Roche, Mannheim, Germany) for 15 min at 4°C. Next, 50 μg of total protein was loaded onto a 12% SDS-PAGE gel and transferred onto polyvinylidene fluoride membranes. Then, the membranes were incubated with the primary antibodies listed in Table 1. Next, the membranes were incubated for 2 h at room temperature with the horseradish peroxidase (HRP)-conjugated secondary antibodies listed in Table 1. Finally, protein bands were visualised using the Immobilon Western Chemiluminescent HRP Substrate (Millipore Corporation, Billerica, MA, USA) with a light-sensitive film (Hyperfilm; Amersham Biosciences, Piscataway, NJ, USA).

### 2.10. CFSE-Labelling Assay

Lymphocyte samples obtained from healthy human volunteers were treated as previously reported [17]. The proliferation of lymphocytes was stimulated by means of phytohaemagglutinin (PHA) for 48 h. Then, they were treated with quercetagetin 188 and 282 mM for 24 h. The cultured cells were then harvested, washed twice with PBS, fixed with 2% paraformaldehyde and analysed with a CytoFLEX LX (Beckman Coulter, USA), acquiring a minimum of 20,000 events from each sample. Data analysis was performed using CellQuest software (Becton–Dickinson, Franklin Lakes, NJ, USA).

### 2.11. Statistical Analysis

Results are presented as the mean ± standard error (SEM). Statistical analysis was conducted using Excel software (Microsoft, Seattle, WA, USA). Data are representative of at least three independent assays. One-way analysis of variance (ANOVA) was performed to identify significant differences between the groups. *p* values of < 0.05 were considered statistically significant.

Immunodetected proteins were quantified using the “Analyze” tool in ImageJ software (National Institutes of Health, Bethesda, MD, USA). The number of positive cells and their fluorescence intensity were quantified using digital images from at least three independent assays. Differences between the two groups were identified with one-way ANOVA followed by Tukey's post hoc test for multiple comparisons. *p* values of < 0.05 were considered statistically significant.

## 3. Results

### 3.1. Antiproliferative Effects of Quercetagetin

**Figure 1:**
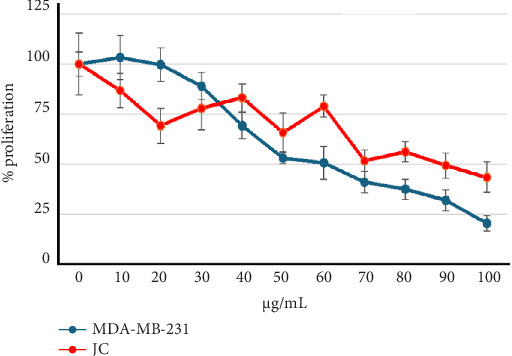
The dose-dependent responses of MDA-MB-231 and JC breast cancer cell lines treated with quercetagetin for 24 h. The data are representative of at least three different assays.

Crystal violet staining [18] was used to assess the antiproliferative effects of quercetagetin on human (MDA-MB-231) and mouse (JC) breast cancer cells. Quercetagetin induced dose-dependent responses (Figure 1). The calculated IC_50_ was 60 μg/mL (188 μM) for MDA-MB-231 cells and 90 μg/mL (282 μM) for JC cells (Table 2).

### 3.2. Necrotic Effect of Quercetagetin

**Figure 2:**
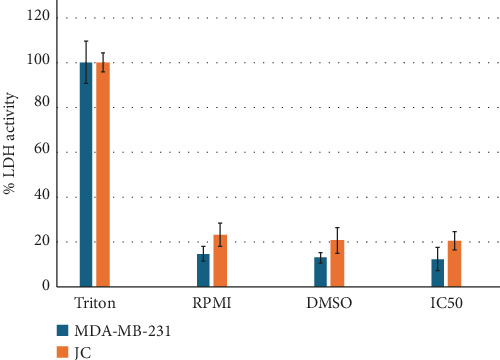
The percentage of LDH activity in cell culture supernatants. The data are presented as the mean of at least three independent assays.

The cell lines were treated with quercetagetin at their respective IC_50_s (MDA-MB-231: 60 μg/mL [188 μM]; JC: 90 μg/mL [282 μM]), and LDH activity was evaluated to determine if it induces necrosis. Triton X-100 (10%) was used as the positive control; this treatment disrupts the cellular membrane, releasing cytoplasmic LDH into the extracellular space and facilitating its detection in a cell culture supernatant. Treatment with quercetagetin did not induce significant necrosis in the breast cancer cell lines compared to the positive and vehicle (DMSO) controls (Figure 2).

### 3.3. Morphological Alterations Induced by Quercetagetin

Since necrosis was not responsible for the observed decrease in cell number, the cells' morphological and biochemical behaviours were analysed to determine which cell death pathways were involved.

**Figure 3:**
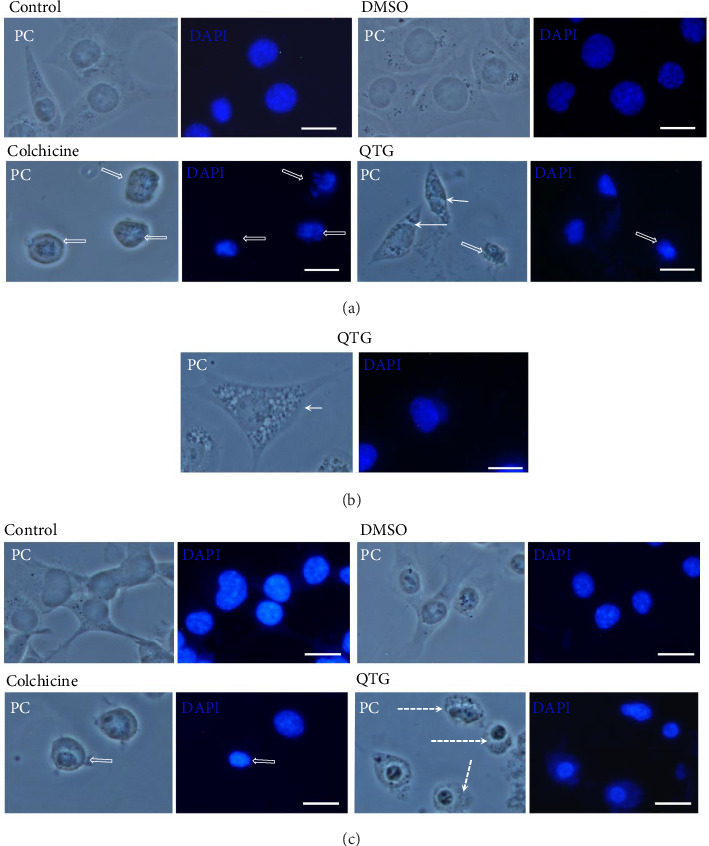
The morphology of human (MDA-MB-231) and mouse (JC) breast cancer cells following quercetagetin treatment. (a, b) Human MDA-MB-231 and (c) mouse JC cells. Under the untreated control and DMSO-treated conditions, both cell lines exhibited regular morphologies. However, after treatment with the positive control (colchicine, known to induce apoptosis), both cell lines exhibited compacted morphologies. After treatment with the flavonoid quercetagetin, both cell lines exhibited diverse morphologies, with some cells showing a compact morphology (empty arrows), suggesting apoptosis. The MDA-MB-231 cells (a, b) showed rounded cytoplasmic vesicles (arrows), whereas the JC cells (c) exhibited enlarged vacuoles (dotted arrow). Scale bars = 20 μm.

Light microscopy of the human (MDA-MB-231) and mouse (JC) breast cancer cell lines revealed different morphologies, corresponding to the activation of distinct cell death pathways. Specifically, some of the MDA-MB-231 cells exhibited apoptotic characteristics such as cell compaction, DNA fragmentation and blebbing (Figure 3(a)). However, several other cells exhibited cytoplasmic extension and the increased formation of rounded vacuoles, but no nuclear alterations, suggesting autophagy (Figure 3(b)). In contrast, while a few murine JC cells exhibited an apoptotic morphology, they predominantly showed elongated cytoplasmic vesicles, suggesting the activation of a process distinct from both apoptosis and autophagy (Figure 3(c)).

### 3.4. Identification of Apoptosis Induction

**Figure 4:**
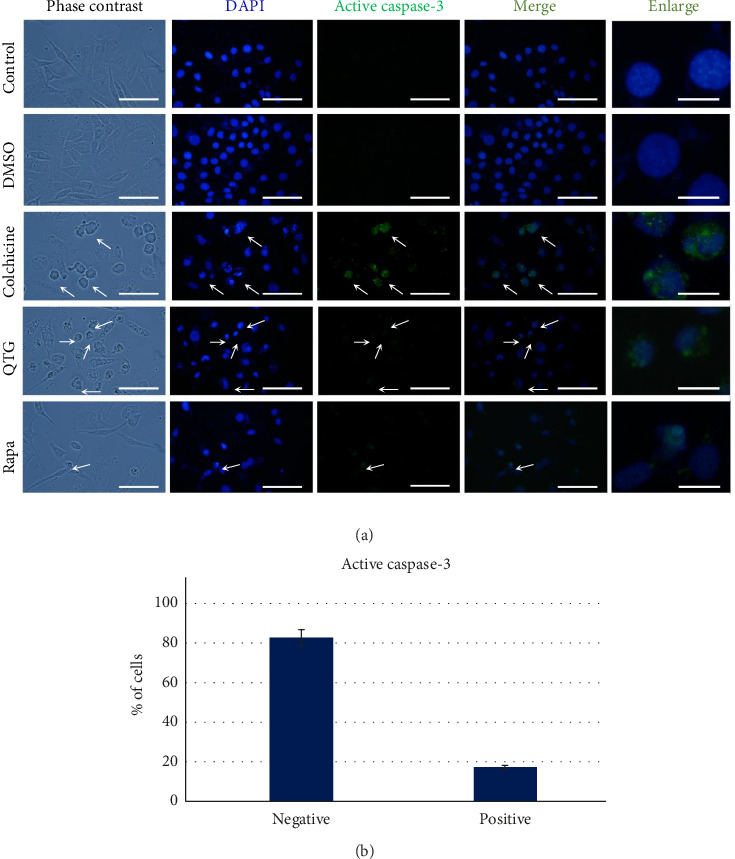
Immunocytochemistry of proapoptotic active caspase-3 in human MDA-MB-231 breast cancer cells. After treatment with quercetagetin, 18% of the cells were caspase-3-positive (b), indicating that only some cells were eliminated by apoptosis (a). Rapamycin was used as a positive control to the autophagy process. Arrows denote apoptotic cells. The data are presented as the mean ± SEM of three independent assays with six technical replicates each. Scale bars = 50 μm (standard) or 20 μm (enlarged).

**Figure 5:**
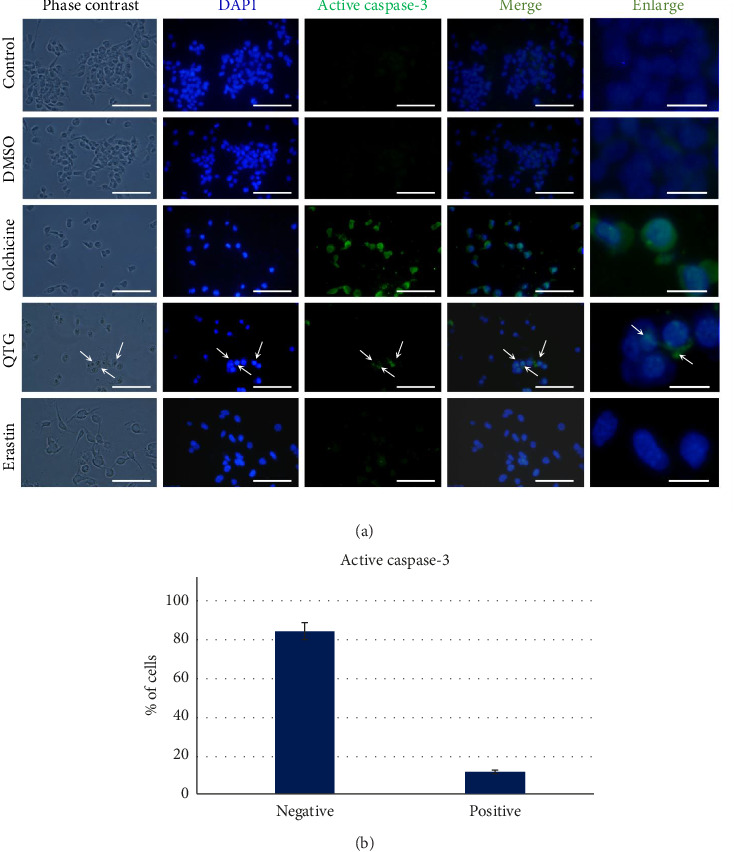
Immunocytochemistry of proapoptotic active caspase-3 in mouse JC breast cancer cells (a). 17% of cells treated with quercetagetin were active caspase-3-positive (b). Erastin was used as a positive control to the ferroptosis process. Arrows denote apoptotic cells. The data are presented as the mean ± SEM of three independent assays with six technical replicates each. Scale bars = 50 μm (standard) or 20 μm (enlarged).

Active caspase-3 was detected using immunocytochemistry to identify apoptosis induction. Following treatment with quercetagetin, fewer than 20% of cells from each cell line expressed caspase-3 (Figures 4 and 5), indicating that quercetagetin induced apoptosis in only a subset of cells (Figures 4 and 5). MDA-MB-231 human cells treated with the proapoptotic colchicine exhibited cytoplasmic positive labelling for active caspase-3, similar to the subset of cells treated with quercetagetin (Figure 4). Similarly, few JC mouse cells were positive for active caspase-3, evidencing that quercetagetin exerts a low proapoptotic effect in this cancer cell line (Figure 5).

### 3.5. Ultrastructural Characterisation

Our morphologic and immunocytochemical data indicated that only a small proportion of cells from both lines undergo apoptotic cell death following treatment with quercetagetin, suggesting the involvement of an alternate cell death pathway. Therefore, the cells' ultrastructure was analysed to define the nature of their cytoplasmic vesicles.

**Figure 6:**
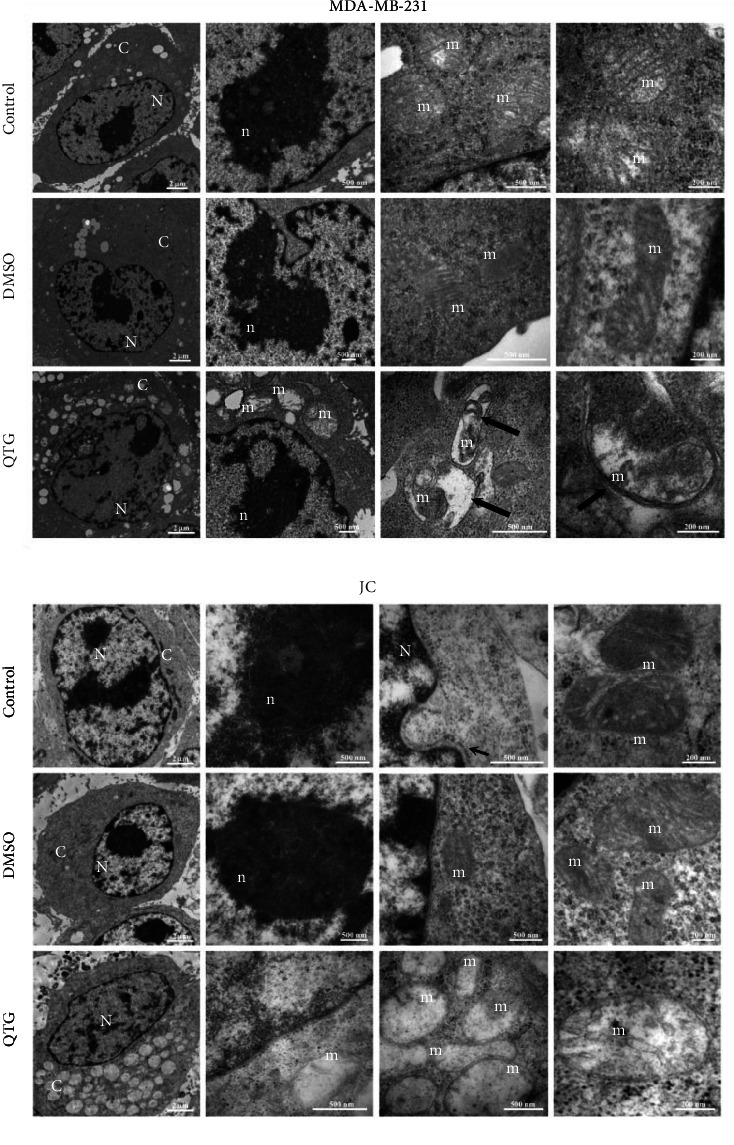
Ultrastructural visualisation of human (MDA-MB-231) and mouse (JC) breast cancer cell lines. Untreated cells show nuclei (N) occupying an ample cytoplasmic space (C). Their nucleoli (n) are large, but their mitochondria (m) have normal morphologies with clearly distinguishable cristae. Treated MDA-MB-231 cells showed vesicles containing mitochondria at different stages of degradation. Treated JC cells showed mitochondria with highly dilated cristae, suggesting the activation of ferroptosis.

Ultrastructural analysis of the untreated and DMSO-treated conditions revealed that both cell lines had large nuclei and nucleoli that extended into the nucleoplasm (Figure 6). Mitochondria were easily identifiable in the cytoplasm. After treatment with quercetagetin, the cells exhibited altered mitochondrial morphology. Specifically, the MDA-MB-231 cells contained autophagic vesicles filled with damaged mitochondria, indicating the activation of autophagy. In contrast, JC cells contained extremely dilated mitochondria, indicating that the cristae were destabilised after treatment with quercetagetin. Notably, the cells' nuclear spaces were unaffected. These data suggest that quercetagetin induces ultrastructural changes related to autophagy in MDA-MB-231 cells but provokes ferroptosis in JC cells.

### 3.6. Evaluation of Autophagy Induction

Based on these findings, we then assessed mitochondrial and lysosomal function in both cancer cell lines to further characterise the autophagy process.

**Figure 7:**
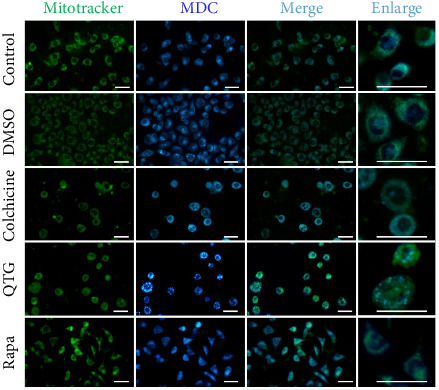
Mitochondria and lysosomes in human (MDA-MB-231) breast cancer cells. Untreated control and vehicle (DMSO)-treated cells showed mitochondria and lysosomes amply distributed in the cytoplasmic space. Quercetagetin (QTG)- and rapamycin (Rapa)-treated cells showed colocalised mitochondria and lysosomes. Rapamycin was used as a positive control to autophagy. Scale bars = 50 μm.

Mitochondria and lysosomes were detected simultaneously to determine whether autophagy was induced by quercetagetin. Human (MDA-MB-231) breast cancer cells treated with quercetagetin showed greater MDC labelling than the untreated, DMSO-treated and colchicine-treated cells (Figure 7). The same effect was observed in cells treated with the positive control, rapamycin, which is known to induce autophagy. Notably, the lysosomal (MDC) and mitochondrial (MitoTracker) labels colocalised in MDA-MB-231 cells treated with quercetagetin, supporting the induction of mitochondrial degradation (Figure 7).

**Figure 8:**
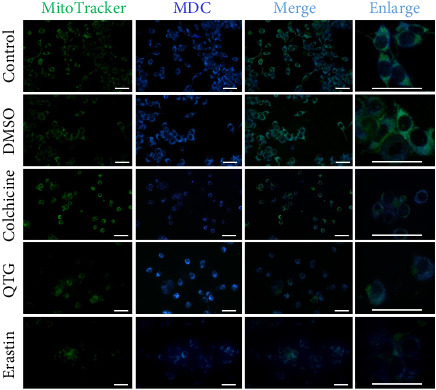
Mitochondria and lysosomes in the mouse (JC) breast cancer cell line. Upon labelling, both organelles were distributed in the cytoplasm of the untreated control and vehicle (DMSO)-treated cells. Quercetagetin (QTG)- and erastin-treated cells showed similar distributions of mitochondrial and lysosomal labelling, although mitochondrial labelling was scarce. Erastin treatment was used as a positive control to ferroptosis. Scale bars = 50 μm.

In contrast, mouse (JC) breast cancer cells treated with quercetagetin showed no MitoTracker mitochondrial labelling. However, agglutinated MDC labelling in the cytoplasm was observed (Figure 8). While these observations suggest mitochondrial dysfunction, these mitochondria were not degraded via autophagy. Indeed, similar results were obtained with the ferroptosis inducer erastin (Figure 8).

### 3.7. Detection of Autophagy Induction

After observing autophagy, the autophagy-related proteins lysosomal-associated membrane protein 1 (LAMP1) and LC3 (also known as microtubule-associated protein 1 light chain 3 alpha; MAP1LC3A) were detected via immunocytochemistry to confirm its activation in response to quercetagetin.

**Figure 9:**
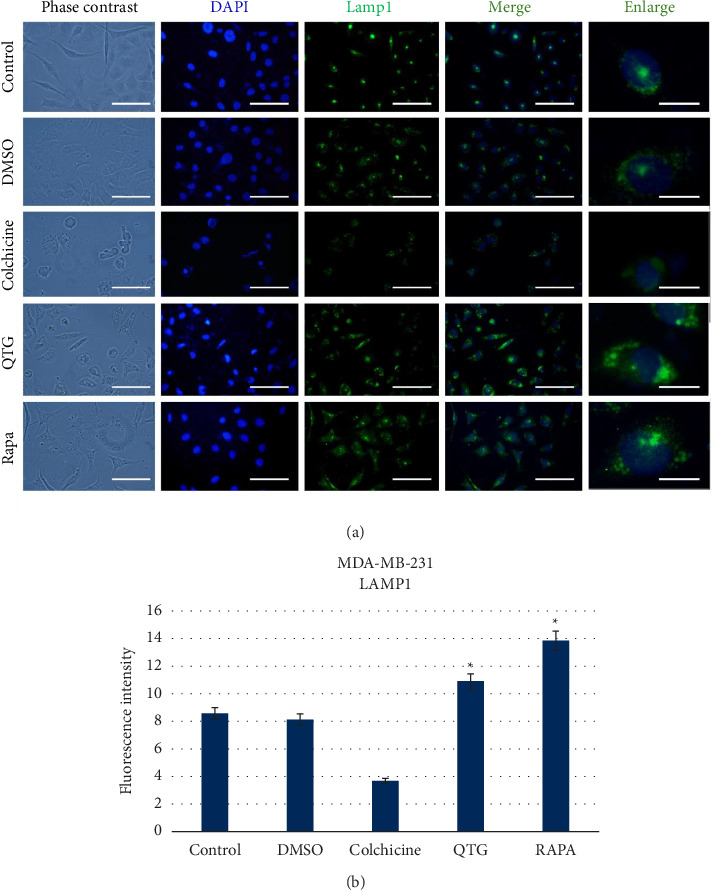
Immunocytochemical detection of the autophagic protein LAMP1 in human (MDA-MB-231) breast cancer cells (a). (b) Graphical representation of the levels of fluorescence intensity in different experimental conditions. The data are presented as the mean ± SEM of three independent assays with six technical replicates each. Scale bars = 50 μm (standard) or 20 μm (enlarged).

**Figure 10:**
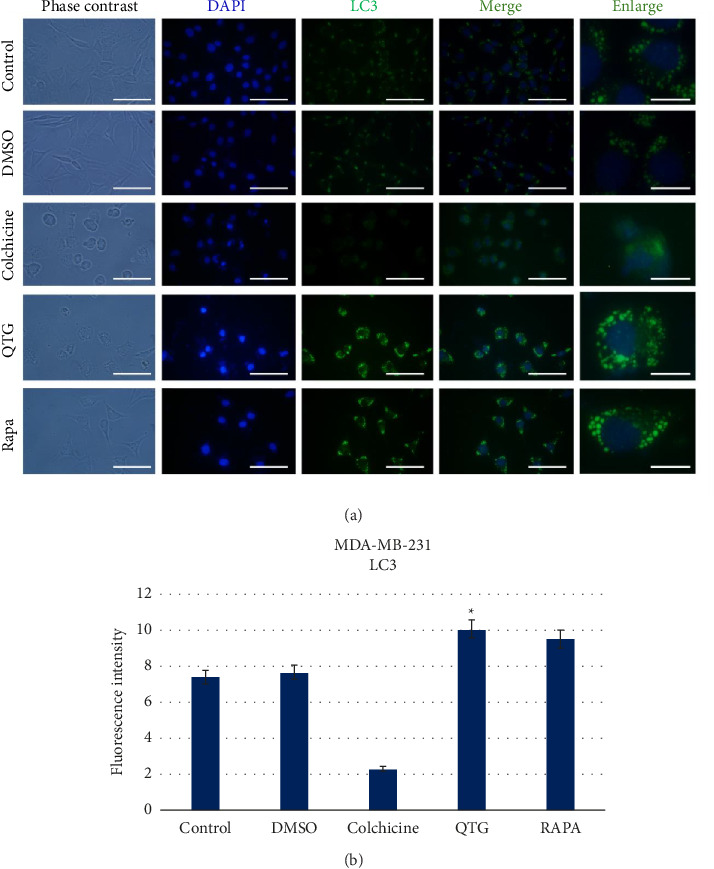
Immunocytochemical detection of the autophagic protein LC3 in human (MDA-MB-231) breast cancer cells (a). (b) Graphical representation of the levels of fluorescence intensity corresponding to LC3 protein labelling. The data are presented as the mean ± SEM of three independent experiments with six technical replicates each. Scale bars = 50 μm (standard) or 30 μm (enlarged).

Human (MDA-MB-231) and mouse (JC) breast cancer cells showed different responses to quercetagetin treatment. Specifically, LAMP1 labelling in round cytoplasmic vesicles was significantly greater in quercetagetin-treated than in control MDA-MB-231 cells (Figure 9(a)), indicating increased autophagic activity (Figure 9(b)). Similarly, LC3 labelling (Figure 10(a)) was greater in quercetagetin-treated than in control cells (Figure 10(b)). The intensity of positive LAMP1 and LC3 labelling was similarly increased in cells treated with the autophagy inducer rapamycin, a positive control.

**Figure 11:**
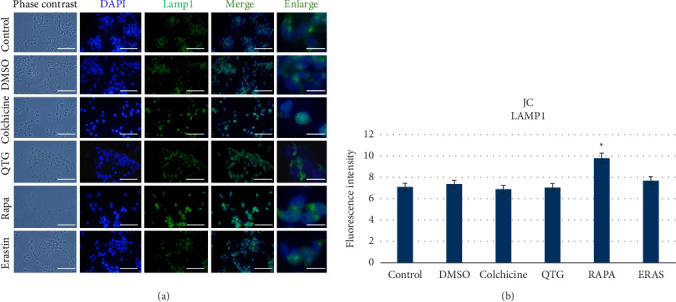
Immunocytochemical detection of the autophagic protein LAMP1 in mouse (JC) breast cancer cells (a). (b) Graphical representation of the levels of fluorescence intensity in different experimental conditions. The data are presented as the mean ± SEM of three independent assays conducted in sextuplicate. Scale bars = 50 μm (standard) or 20 μm (enlarged).

**Figure 12:**
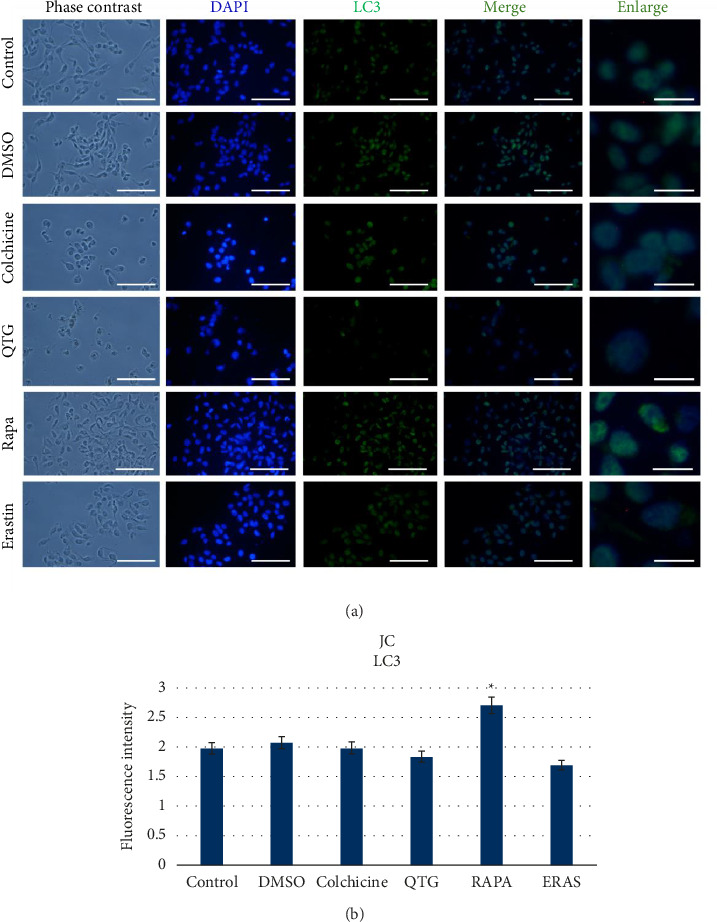
Immunocytochemical detection of the autophagic protein LC3 in mouse (JC) breast cancer cells (a). (b) Graphical representation of the levels of fluorescence intensity corresponding to LC3 protein labelling. The data are presented as the mean ± SEM across three independent assays performed in sextuplicate. Scale bars = 50 μm (standard) or 20 μm (enlarged).

In contrast, when mouse (JC) breast cancer cells were treated with quercetagetin, the immunocytochemical labelling of the autophagic proteins LAMP1 and LC3 did not increase significantly (Figures 11 and 12). However, LAMP1 and LC3 labelling did increase after rapamycin treatment.

Altogether, these results indicate that JC cells treated with quercetagetin do not undergo apoptosis and/or autophagic cell death. Taken together with our ultrastructural characterisation data, these observations suggest that quercetagetin induces ferroptosis instead.

### 3.8. Lipid Peroxidation Analyses

**Figure 13:**
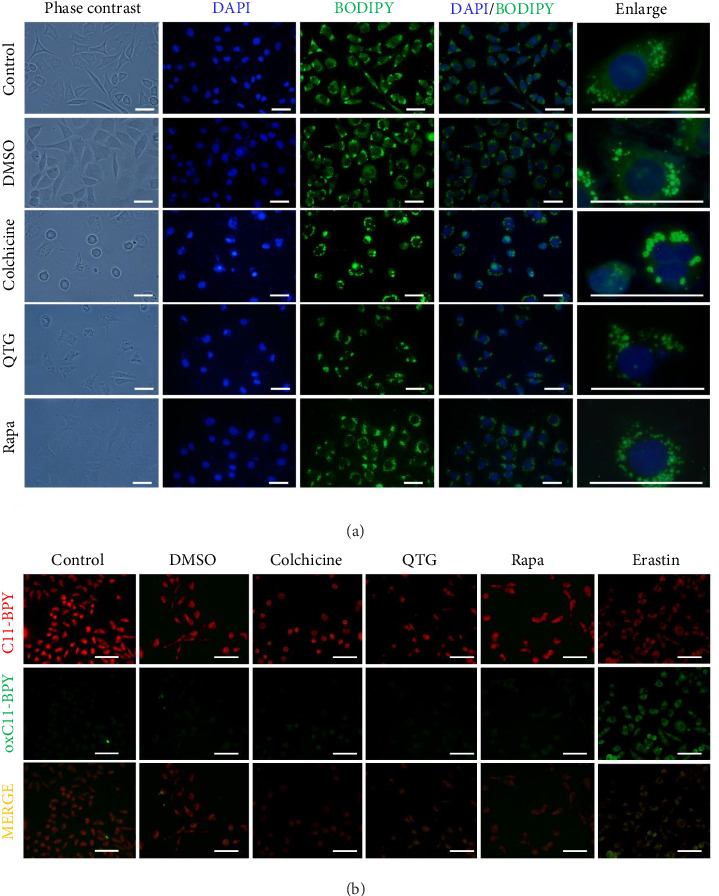
Labelling of neutral and peroxidised lipids in human (MDA-MB-231) breast cancer cells. (a) Neutral lipids were distributed in the cytoplasmic space under all experimental conditions except for apoptosis (colchicine). (b) Peroxidised lipids were present only in cells treated with erastin. Scale bars = 50 μm.

**Figure 14:**
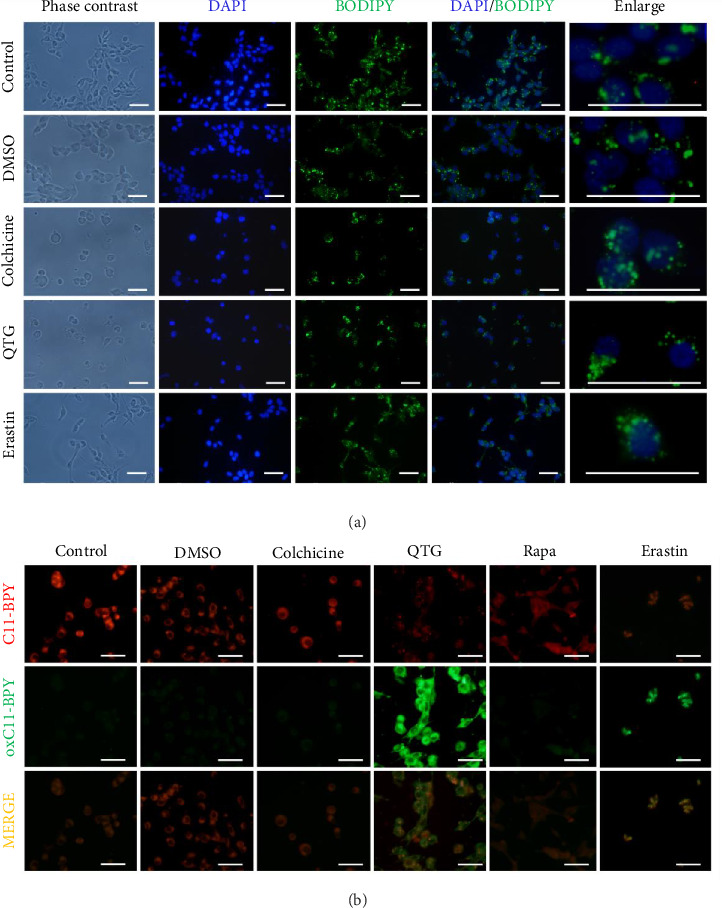
Labelling of neutral and peroxidised lipids in mouse (JC) breast cancer cells. (a) Neutral lipids were distributed in the cytoplasmic space. (b) Peroxidised lipids were present in cells treated with quercetagetin (QTG) and erastin. Scale bars = 50 μm.

Neutral and peroxidised lipids were analysed to determine whether quercetagetin induces ferroptosis in breast cancer cells. The presence and distribution of neutral lipids were assessed in both human (MDA-MB-231) and mouse (JC) cancer cells treated with quercetagetin using BODIPY labelling. Neutral lipids were distributed in the cytoplasmic space (Figures 13(a), 14(a)), changing only in cells treated with colchicine (positive control for apoptosis). In contrast, peroxidised lipids increased in JC cells treated with quercetagetin. Similar effects were observed in MDA-MB-231 and JC cells treated with erastin (Figures 13(b), 14(b)). Notably, MDA-MB-231 cells did not show increased labelling corresponding to the presence of peroxidised lipids (Figure 13(b)).

### 3.9. Western Blotting Analysis

**Figure 15:**
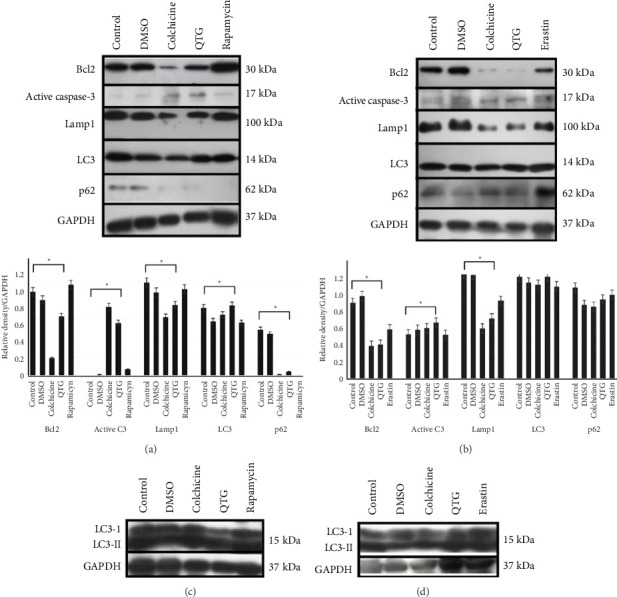
Immunoblots of apoptosis- and autophagy-related proteins in human (MDA-MB-231) and mouse (JC) breast cancer cells treated with quercetagetin. The levels of apoptotic and autophagic proteins in (a) MDA-MB-231 and (b) JC cells. (c) and (d) LC3-I conversion and LC3-II turnover to MDA-MB-231 and JC , respectively. The data were compared to those of control cells in at least three independent assays (^∗^). All data are expressed as the mean ± SEM.

Immunoblotting of proteins related to autophagy and apoptosis confirmed that quercetagetin induced apoptosis and autophagy in human (MDA-MB-231) breast cancer cells, consistent with our ultrastructural and immunocytochemical experiments. Specifically, we observed decreased expression levels of BCL2 and the presence of active caspase-3, confirming that quercetagetin induces apoptosis. We also observed increased levels of the proautophagic protein LC3-II, as well as decreased p62/sequestosome (SQSTM1) expression, supporting the induction of autophagy in MDA-MB-231 cells (Figure 15(a)). Conversely, among the proteins related to autophagy and apoptosis, only the levels of BCL2 changed significantly in JC cells treated with quercetagetin (Figure 15(b)). Thus, JC cells do not die by autophagy or apoptosis. Additionally, the LC3-I to LC3-II conversion evidenced the autophagic activity induced by quercetagetin in MDA-MB-231 cells (Figure 15(c)). Differently, JC did not show changes in the converted LC3-II protein, indicating that quercetagetin is not exerting an autophagic effect in this cellular type (Figure 15(d)).

### 3.10. Quercetagetin Selectivity

**Figure 16:**
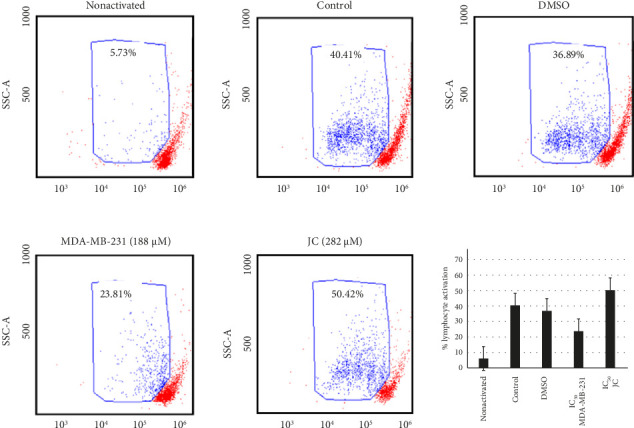
Selective effect of quercetagetin on noncancerous lymphocytes. Lymphocytes from ostensibly healthy donors were treated with quercetagetin after activation. The data were compared to those from activated control lymphocytes in at least three independent assays. All data are expressed as the mean ± SEM.

After establishing that quercetagetin induced different forms of cell death in human (MDA-MB-231) and mouse (JC) breast cancer cells, the effects of this compound on normal lymphocyte activation were evaluated to assess its selectivity. When lymphocytes were treated with quercetagetin at concentrations corresponding to the IC_50_s for MDA-MB-231 and JC cells, their activation was not significantly affected (Figure 16), indicating a degree of compound selectivity.

## 4. Discussion

Breast cancer is a significant public health concern worldwide. Many efforts have been directed towards identifying new therapeutic strategies for breast cancer, including exploring the anticancer properties of compounds from natural products, such as those from the plant kingdom. In this study, we explored the effects of quercetagetin isolated from *Tagetes erecta* on human (MDA-MB-231) and murine (JC) breast cancer cells. We observed that quercetagetin induced antiproliferative effects in both breast cancer cell lines with IC_50_ of 60 μg/mL (188 μM) and 90 μg/mL (282 μM), respectively. Evidence from LDH activity suggested that the observed reduction in the cell number was not due to necrosis, which was not induced to significant levels in either cell line. Necrosis involves the disruption of plasma membranes, leading to tissue damage and generating a key inflammatory response [19]; programmed cell death elicits a different response. During chemotherapy, necrosis is associated with side effects; the nonnecrotic antitumoural effect of quercetagetin is therefore an advantage, potentially promoting the control of cellular proliferation with reduced side effects and improved tolerance.

As we have previously reported, quercetagetin induces the expression of proteins related to apoptosis [8], a form of programmed cell death regulated by caspase proteases and members of the BCL2 family, such as BCL2-associated X apoptosis regulator (BAX), BCL2 antagonist/killer 1 (BAK1/BAK) and BCL2 itself [20, 21]. Morphologically, apoptosis is characterised by cellular and nuclear compaction, cytoplasmic blebbing, DNA fragmentation and apoptotic body formation [11]. Quercetagetin induced apoptosis in a small proportion of our cells (approximately 20%), evident in morphological results and supported by the detection of active caspase-3 and decreased BCL2 expression in both human (as previously reported [8]) and murine (JC) breast cancer cells. Together these results indicate that quercetagetin triggers apoptosis through downregulation of the antiapoptotic protein BCL2 and subsequent activation of executioner caspase-3. Once activated, caspase-3 acts inside the intracellular substrates causing the morphological changes observed in treated cells. Interestingly, we also identified treated cells with cytoplasmic vesicles which were not positive for active caspase-3. To explore this finding, we investigated the mechanisms of cell death in these populations in greater depth.

There are several well-known forms of programmed cell death, including autophagy and the more recently described ferroptosis [22]. Autophagy is a conserved catabolic process that recycles intracellular contents and eliminates damaged organelles [23, 24]. The role of autophagy in cell death is well-documented; it is characterised by the presence of cargo-laden autophagosomes in different stages of degradation and is regulated by ATG proteins and high lysosomal activity [25, 26]. The ATG8/LC3 family consists of important autophagy-related proteins, and LC3 itself is a common marker for identifying autophagosomes [26]. LC3 is converted into LC3-II by means of a lipidation process with phosphatidylethanolamine (PE); both forms are present in the autophagosome membrane [27, 28].

We found that quercetagetin treatment increased LC3 labelling in MDA-MB-231 cells, indicating induced autophagy. Furthermore, the presence of the lapidated form, LC3-II, was significantly increased in quercetagetin-treated cells, further suggesting an increase in the autophagic process. In addition to the ultrastructural observations showing abundant autophagic-autophagolysosomal vesicles containing mitochondria, these findings support the notion that quercetagetin induces cell death via the autophagy pathway. Indeed, the downregulation of SQTSM1/p62 indicates active cytoplasmic autophagosome elimination [26, 29]. In contrast, quercetagetin did not increase autophagy marker levels in JC cells. Moreover, our ultrastructural observations in these cells highlighted morphological changes associated with different cell death processes, including those indicative of both autophagy and ferroptosis. These data were further supported by the presence of mitochondrial damage [22, 30] and lipid peroxidation [31], both of which are associated with ferroptosis. From these observations, we propose that in MDA-MB-231 human breast cancer cells, quercetagetin induces sustained, irreparable mitochondrial damage; SQSTM1/p62 labelling leads to the activation of autophagic degradation of the organelle and ultimately to cell death. Meanwhile, in JC mouse breast cancer cells, quercetagetin provokes lipid peroxidation, which induces mitochondrial alterations related to a ferroptosis-like process.

Breast cancer is the leading cause of death in women worldwide. Identifying new therapeutic strategies to prevent breast cancer progression is crucial. Herein, we demonstrate that quercetagetin, isolated from *T. erecta*, exhibits antiproliferative activity in breast cancer cell lines of human and mouse origin. We provide evidence for the induction of multiple known forms of programmed cell death, specifically apoptosis, autophagy and ferroptosis [22, 32, 33]; the cell lines exhibited distinct responses to treatment. Importantly, human MDA-MB-231 triple-negative breast cancer cells exhibited mitochondrial damage, indicating activation of autophagy. In contrast, the murine JC breast cancer cell line underwent ferroptosis-like cell death. Notably, quercetagetin did not induce significant necrosis, enhancing its anticancer properties. The efficacy of chemotherapy relies on the cytotoxic effects of compounds frequently associated with necrosis [34–36]. The induction of multiple modes of cell elimination in tumour cells by quercetagetin indicates a moderate immune response when compared with necrosis-inducing compounds. Furthermore, the assays with nontumour cells evidence that quercetagetin does not inhibit the activation of noncancerous lymphocytes, a cell population highly affected by conventional chemotherapies [37–39]. Taken together, these results highlight the potential of quercetagetin in cancer treatment. Its capacity to activate different programmed cell death routes may promote more efficient cellular control and reduce side effects. The present study provides evidence which may contribute to the development of new strategies for cancer cell control, potentially alleviating the side effect burden of treatments that rely on necrotic cell death.

Our findings indicate that quercetagetin induces different types of programmed cell death in human and mouse tumour cells, without triggering significant necrotic activity. This suggests that it may produce minor necrosis-related side effects when compared with conventional chemotherapeutics. However, human tumour cells were preferentially eliminated through autophagy, while ferroptosis was predominantly induced in mouse cells. This suggests differential, species-specific responses of human and mouse tumour cells to quercetagetin, although apoptosis may be a common mode of cell death. These results are consistent with the fact that tumours comprise different cell types, including tumour stem cells, which maintain tumour growth and confer drug resistance [40–42] and cells compromised by tumour growth [43, 44]. This opens the possibility that both cell types may be eliminated differentially but through a common pathway of programmed cell death.

## 5. Conclusion

Our present results and previous observations [8] demonstrate the potential of the flavonoid quercetagetin from *Tagetes erecta* as an anticancer agent. It exerts antiproliferative effects, induces different forms of programmed cell death, has low necrotic potential and exhibits important selectivity.

## Figures and Tables

**Table 1:** List of antibodies used in the study.

Primary antibodies	Host species	Supplier	Assay/dilution
Lamp1	Rabbit	Abcam Cat.: ab24170	1/100 Immunodetection 1/2500 WB
LC3	Rabbit	Thermo Fisher Cat.: PA116930	1/100 Immunodetection 1/2500 WB
Cas3A	Rabbit	Sigma-Aldrich Cat.: C8487	1/100 Immunodetection 1/2500 WB
p62	Rabbit	Abcam Cat.: ab155686	1/2000 WB
BCL2	Mouse	Sigma-Aldrich Cat.: B9804	1/2500 WB
GAPDH	Rabbit	Millipore Cat.: ab516	1/10,000 WB

**Table 2:** IC_50_s of quercetagetin for MDA-MB-231 and JC breast cancer cells.

Cell line	IC_50_ (μg/mL)	IC_50_ (µM)
MDA-MB-231	60	188
JC	90	282

## Data Availability

The data are available from the first author upon request.
